# Chemotaxis: A Feedback-Based Computational Model Robustly Predicts Multiple Aspects of Real Cell Behaviour

**DOI:** 10.1371/journal.pbio.1000618

**Published:** 2011-05-17

**Authors:** Matthew P. Neilson, Douwe M. Veltman, Peter J. M. van Haastert, Steven D. Webb, John A. Mackenzie, Robert H. Insall

**Affiliations:** 1 Cancer Research UK Beatson Institute, Glasgow, United Kingdom; 2 Department of Cell Biochemistry, University of Groningen, Groningen, The Netherlands; 3 Department of Mathematics and Statistics, University of Strathclyde, Glasgow, United Kingdom; Yale University, United States of America

## Abstract

The mechanism of eukaryotic chemotaxis remains unclear despite intensive study. The most frequently described mechanism acts through attractants causing actin polymerization, in turn leading to pseudopod formation and cell movement. We recently proposed an alternative mechanism, supported by several lines of data, in which pseudopods are made by a self-generated cycle. If chemoattractants are present, they modulate the cycle rather than directly causing actin polymerization. The aim of this work is to test the explanatory and predictive powers of such pseudopod-based models to predict the complex behaviour of cells in chemotaxis. We have now tested the effectiveness of this mechanism using a computational model of cell movement and chemotaxis based on pseudopod autocatalysis. The model reproduces a surprisingly wide range of existing data about cell movement and chemotaxis. It simulates cell polarization and persistence without stimuli and selection of accurate pseudopods when chemoattractant gradients are present. It predicts both bias of pseudopod position in low chemoattractant gradients and—unexpectedly—lateral pseudopod initiation in high gradients. To test the predictive ability of the model, we looked for untested and novel predictions. One prediction from the model is that the angle between successive pseudopods at the front of the cell will increase in proportion to the difference between the cell's direction and the direction of the gradient. We measured the angles between pseudopods in chemotaxing Dictyostelium cells under different conditions and found the results agreed with the model extremely well. Our model and data together suggest that in rapidly moving cells like Dictyostelium and neutrophils an intrinsic pseudopod cycle lies at the heart of cell motility. This implies that the mechanism behind chemotaxis relies on modification of intrinsic pseudopod behaviour, more than generation of new pseudopods or actin polymerization by chemoattractants.

## Abstract 2

A simple feedback model of chemotaxis explains how new pseudopods are made and how eukaryotic cells steer toward chemical gradients.

## Author Summary

The efficiency, sensitivity, and huge dynamic range of eukaryotic cell chemotaxis have proven very hard to explain. Cells respond to shallow gradients of chemotactic molecules with directed movement, but the mechanisms remain elusive. Most current models predict that cells have an internal “compass” produced by processing the extracellular signal into an intracellular mechanism that points the cell towards the gradient and steers it in that direction. In this article, we present evidence that this internal compass does not exist; instead, the cell orients itself simply by making use of its pseudopods—the dynamic finger-like projections on the surface of the cell. We approached the question by making a computational model of the movement of a cell without a compass. In this model, the cell moves in a convincingly natural way simply by using its pseudopods, which respond to positive- and negative-feedback loops. The concentration of the chemoattractant molecule modulates the amount of positive feedback. Apart from this, no signal processing is necessary. This simple model reproduces many observations about normal chemotaxis. It also accurately predicts the angle at which new pseudopods split off from old ones, which had not been previously measured. The computational model thus demonstrates that pseudopod-based mechanisms are powerful enough to explain chemotaxis.

## Introduction

**Figure 1:**
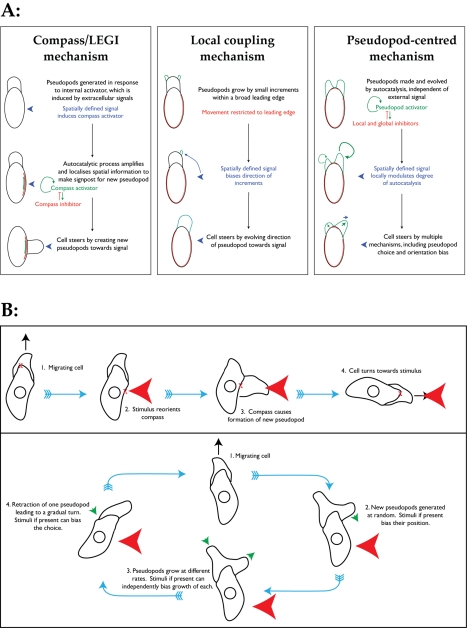
Comparison of different mechanisms. (A) Comparison of the underlying ideas behind different mechanisms that have been proposed to explain chemotaxis. Compass and LEGI mechanisms emphasize signal processing to determine the correct site for pseudopod generation (the “compass needle”); the local coupling mechanism restricts protrusion to a leading edge and uses the attractant to bias the growth of different parts of the leading edge; the pseudopod-centred mechanism emphasises the endogenous, autocatalytic growth of pseudopods and allows the unprocessed gradient information to bias multiple points in the cycle. (B) The pseudopod cycle and pseudopod-centred mechanisms. In the traditional signal-centred view, the cell forms an internal representation of the gradient (the compass) that directs the formation of new pseudopods. The compass can only affect the process at one point. Pseudopod-centred views hold that the generation and evolution of pseudopods is driven by cyclical internal processes, and when present chemoattractants bias multiple different steps in the cycle.

Eukaryotic chemotaxis—cell migration towards a source of attractants—is both biologically important and theoretically interesting, so it has been widely studied. Recently, a majority of authors have considered that chemotaxis is driven by a “compass” [1]. The exact meaning of the compass varies. When originally defined [2], it implied that there is a simple “compass needle” inside the cell, which is a localised signal that represents the direction of the chemoattractant gradient (Figure 1A). If the hypothetical compass needle points in a different direction from the cell's current direction, it causes new pseudopods to be made towards attractant sources, thus steering the cell. More recent compass-based models consider the noisy environment in which chemoattractants are sensed, allowing the compass to bias (rather than specify) the positions of new pseudopods. A number of relatives of compass models (including the LEGI models from the Iglesias and Devreotes groups [3], the balanced inactivation model of Levine [4], and inositide-based models such as Narang [5]) share one property—they focus on information processing, at the level of receptor occupancy and immediately below, giving the cell a simplified and amplified internal message that determines the position and direction of future pseudopods. In these models the cytoskeleton mostly plays a blue-collar role, responding to the instructions from the internal compass.

However, we [6]–[8] and others [9]–[11] have found that simple generation of new pseudopods cannot explain observed cell steering and that, unless gradients are very steep, new pseudopods are usually more strongly controlled by internal dynamics than by chemoattractants. We have therefore proposed a “pseudopod-centred” mechanism (Figure 1A) [12], in which there is no requirement for a compass or other internal messenger representing direction. Rather, each cell's direction is entirely represented by the pseudopods themselves. We have demonstrated that new pseudopods are mainly generated by bifurcation and evolution of existing ones [6],[7]. In a variety of cell types, close to 90% of new pseudopods are generated when existing pseudopods split to form two daughters. This severely limits the place and time at which pseudopods can emerge. We find that directional migration is accomplished by biasing the cycle of pseudopod generation and retraction, at any of several steps, rather than simply at the level of new pseudopod initiation. These include selecting the best of multiple pseudopods generated by random splitting [6] and biasing the position at which new pseudopods emerge [7]. Several other lines of data support this mechanism. For example, new pseudopods on average steer the cell away from the attractant—which disagrees with compass models in which the aggregate effect of new pseudopods is to steer the cell towards the source.

An alternative, groundbreaking way of addressing the same issues uses a “local coupling” model (Figure 1A) [13]. Here the leading edge is restricted to a proportion of the cell and grows by small increments. As with our pseudopod-centred model, chemoattractants bias an internal process and there is no need for signal processing. However, this model has two disadvantages. It is limited to cells like neutrophils with broad, stable leading edges that turn without generating or retracting pseudopods, and thus does not deal well with cells like Dictyostelium or macrophages. Similarly, the process that restricts the pseudopod size and prevents actin polymerization at the sides is central to the model, whereas in many cell types actin may polymerize at any part of the cell [14]. In this work we therefore addressed the pseudopod-centred model as a potential broad or universal model for chemotaxis.

## Results

### A Pseudopod-Centred Computational Model

**Figure 2:**
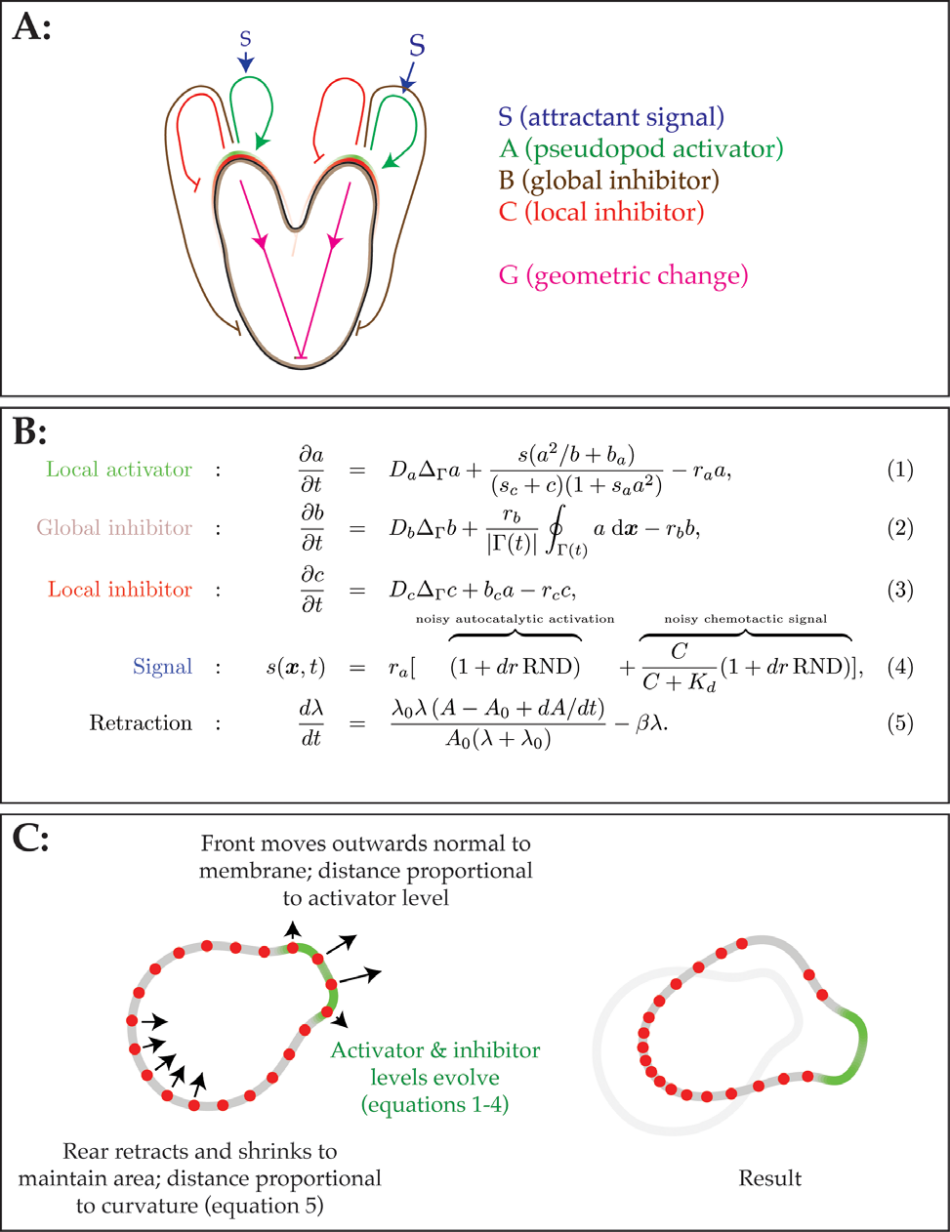
Pseudopod-centred computational model for chemotaxis. (A) Topology of feedback loops. Each pseudopod is driven by a local activator peak (A), which in turn stimulates production of global and local inhibitors (B and C). Coupled expansion of the pseudopods and contraction of the rear also imposes geometric change that can act as an inhibitor by diluting A levels where peaks are expanding. (B) Equations (adapted from Meinhardt [20]). Activator and inhibitor levels change through diffusion, synthesis, and breakdown terms, respectively. The signal is composed of two terms, one representing autocatalysis and the other related to receptor occupancy, each with its own a noise component. (C) Mechanics of model movement. The cell is modelled as a path defined by finite element nodes. The activator and inhibitor levels change according to equations (1)–(4). To move the cell, each element of the perimeter is moved in the outward-normal direction with a velocity that is proportional to the local activator level at that point. Retractions are governed by the local mean curvature of the cell and allow the cell to maintain a roughly constant area over time. The new perimeter—now with unequally spaced nodes—is passed to the level set toolbox to maintain perimeter integrity.

We tested the predictive abilities of pseudopod-centred mechanisms using a conceptually simple computational model, based on coupled feedback loops (Figure 2A). Feedback is fundamental to chemotaxis [15] and underpins both compass- and pseudopod-centred mechanisms but used in different ways. In compass models, feedback is typically invoked during signal processing, to amplify and simplify the noisy and complex information from receptors [16]–[18]. We have predicted that such signal processing is not essential [12],[19]. Rather, in our model feedback loops are used to define the pseudopods themselves. Positive feedback allows pseudopods to maintain themselves and to grow, while negative feedback fulfils two roles—firstly, it restricts the growth of existing pseudopods and the initiation of new ones at other parts of the cell, and secondly, it makes pseudopods dynamic, allowing cells to change shape and direction as occurs in amoeboid movement. To model chemotaxis, we therefore adapted an established system (Figure 1B) [20] based on a single pseudopod activator regulated by three feedback loops, one positive and two negative (Figure 1B). In the Meinhardt article [20], the cell does not move—the components of the feedback loop were localised, in a dynamically evolving pattern, within a static cell perimeter. To allow our simulated cell to move (Figure 1C), we have used an evolving surface finite element method [21], in which each point of the perimeter moves outwards normal to the edge of the cell [22], at a rate proportional to the local activator level. Protrusion is counteracted by a curvature-based contraction, in which the edge is retracted so that the cell tends towards a constant area. The different parts of the cell retract in proportion to their steepness of curvature; this effectively simulates cortical tension, which retracts highly curved areas and thus causes the cell to tend towards a circle. A level set method was used to evolve the cell perimeter. These methods are based on an Eulerian description of a level set function, where the location of the zero level set identifies the cell perimeter [23]. This framework confers many well-known computational advantages, including use of fixed Cartesian meshes and straightforward implementation of high resolution numerical schemes [24]. Full details of the computational methods are given in a separate publication [25].

Importantly, movement of the leading edge greatly changes the evolution of the activator levels, because areas where the level of pseudopod activator is high tend to expand, diluting the activator. Evolution of the edge therefore mimics the local inhibitor, in a way that might make possible a future model with only two further feedback loops, one positive and one negative.

### Physiological Correlates

The centre of our model is a pseudopod activator whose level correlates with the rate of movement of the leading edge. One biologically appropriate equivalent is actin nucleation driven by the Arp2/3 complex, which is a central driver of actin-based movement. However, the components of the model are not intended to directly represent defined molecular species. This is for two reasons. Firstly, the regulation of the actin cytoskeleton is not understood in the quantitative detail needed to generate a defined model. Key components have not been defined or cannot be measured (for example, the affinity of activated Rac for the SCAR/WAVE complex), and multiple factors such as VASP may modulate the rate of actin-based protrusion. Secondly, actin-based motility is frequently regulated by multiple parallel components, so removing individual pathways such as SCAR/WAVE, Rac, or PI 3-kinase does not block migration, despite the clear importance of each of these pathways. Molecule-based models have been successful and informative about individual pathways and the roles of single proteins [26]–[29], but the dynamic morphology of chemotactic cells has proven too complex for such an approach. Our approach is more similar to those successfully used by the Wang, Theriot, and Mogilner labs based initially on cell shape [30] and mechanics [31]. While the activator is directly related to the level of actin nucleation or polymerization, we envisage the local inhibitor corresponding to depletion of required substrates (for example, Arp2/3 complex, activation-competent SCAR/WAVE) and the global inhibitor corresponding to physical processes such as mechanical tension. The positive feedback loop driving pseudopod growth could act at multiple levels, including through Rac [32], SCAR/WAVE [10], or actin itself [33]; all three have been described and probably act concurrently in real cells, though we envisage the first two as being more influential. Again, however, the aim of this model is to test the predictive power of pseudopod-centred models, rather than the roles of particular pathways.

### Modelling Random Migration

**Figure 3:**
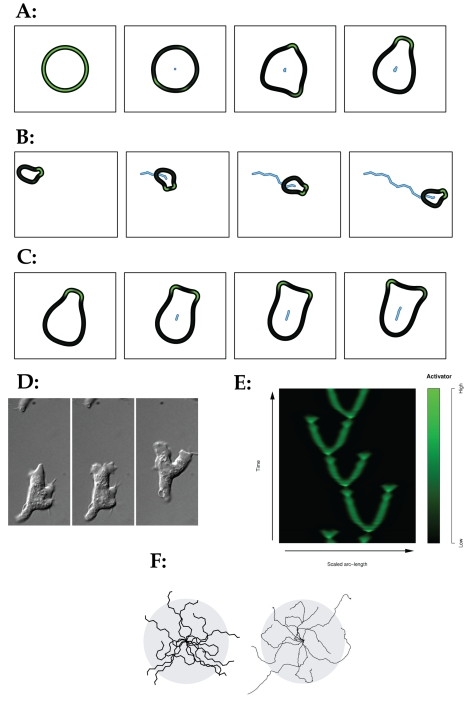
Simulation of random migration without stimulus. (A–D) Initial polarization (A), persistence of migration (B), and pseudopod bifurcation (C) during random migration, compared with DIC images of real migrating *Dictyostelium* cells (D). Black shows cell perimeter; green shows local levels of pseudopod activator; blue shows cell centroid track. (E) Travelling wave patterns in simulated cell perimeters. Perimeters from successive frames were unwrapped from polar to Cartesian (compare with Killich et al. [9]). *x*-axis shows the position on the perimeter, *y*-axis shows the evolution over time, and the colour map indicates the activator level as defined by the adjacent colour profile (with black corresponding to a low activator level, and bright green corresponding to a high activator level). (F) Tracks of several simulated cells (left) compared with real cells (data from [8]). The grey circle indicates the mean dispersal.

The results from this simulation (Movie S1 and Figure 3A–C) make several clear points. Firstly, cells polarize into a front and a rear without needing additional internal signals (Figure 3A). This polarization is seen as an essential part of efficient migration and chemotaxis [17]. Secondly, the simulated cells' migration is persistent—they maintain their direction over several pseudopod cycles (Movie S1 and Figure 3B). Persistence has also been measured in most migrating cells and is thought to be important for chemotaxis [34],[35]. Thirdly, new pseudopods are mostly made by bifurcation of the leading edge (Figure 3C; compare with Dictyostelium cell in Figure 3D). Bifurcation (“pseudopod splitting”) was initially described by Andrew [6] and has since been observed in multiple types of migratory cells [36], including mouse embryonic fibroblasts, human dendritic cells, and cultured neurites. In the measured cell types, the proportion of pseudopods generated by splitting is usually around 90% [6].

Analysis of the positions of pseudopods as they evolve over time also gives a wavelike pattern (Figure 3E), like that measured in real unstimulated cells [9]. Furthermore, the paths taken by individual cells are remarkably similar between the simulation and real cells (Figure 3F), and both display characteristics of a persistent random walk [37]. The simple model based on Meinhardt [20] therefore successfully describes a typical unstimulated cell.

### Modelling Chemotaxis

To generate a pseudopod-centred model of the response to chemoattractant, we departed from the Meinhardt model [20], which relies on hidden signal processing to provide a fully localised signal (see Methods). In our system, the magnitude of the positive feedback is directly correlated with the local chemoattractant receptor occupancy (Figure 2B, equation 4), with additional elements corresponding to noisy signal perception and activator feedback. This provides a key difference between our model (and pseudopod-centred models in general) and most work in the chemotaxis field. In our model, neither actin polymerization nor pseudopod generation is caused by extracellular signals. Rather, the signals are only able to modulate the rates of internal processes. In shallow gradients, internal processes overwhelmingly dominate.

**Figure 4:**
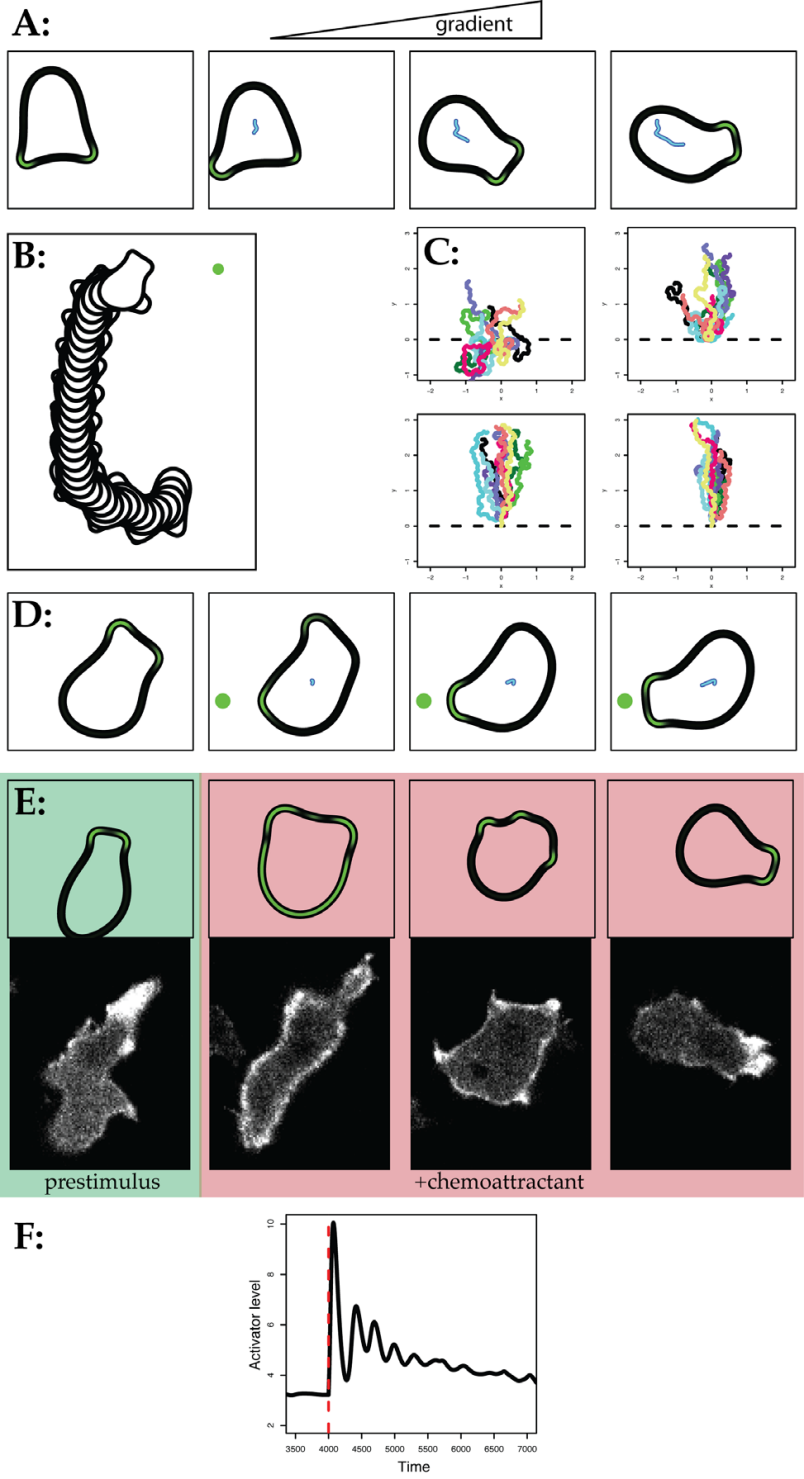
Chemotaxis of simulated cells in shallow and steep gradients. (A) Frames showing a simulated cell reorienting as a chemoattractant gradient is applied. (B) Superimposition of consecutive frames showing reorientation of cell after the chemoattractant source was moved (after frame 7). Compare with Figure 4a from ref. [3]. (C) Tracks of several different simulated cells, corresponding to initial gradients of 5.3 nM – 5.5, 5.7, 6.1, and 6.6 nM across the cell. As with real cells the accuracy increases as the gradient steepens. In each case the chemoattractant gradient is first applied at *t* = 0. (D) Reorientation by de novo pseudopods in a steep gradient. Frames taken from Movie S2, corresponding to a receptor occupancy from 0% to 20% across the cell. (E, F) “Cringe” response to sudden, global increase in chemoattractant concentration from zero to full receptor occupancy. An exponential decay, simulating receptor adaptation, was added to the signal function. (E) shows frames from Movie S4, revealing sequentially an unstimulated cell (green box), global actin polymerization following global stimulation (red box), rounding with bleblike protrusions, and finally recovery and repolarization. A similar cringe response in a real Dictyostelium cell transfected with GFP-lifeact (from Movie S4) and viewed in a confocal microscope is shown below. (F) shows the aggregate activator for a single cell perimeter versus time—compare with the actin curve in [43].

When this connection to external signalling is added and a moderate chemoattractant gradient (from 5.3 nM to 6.5 nM across the cell) is applied, the simulated cell moves very similarly to a real Dictyostelium in a similar gradient (Movie S1; Figures 4A,B). This close resemblance to real cells is surprising, given a number of disagreements with generally accepted points.

Firstly, as previously stated there is no direct connection between the external signal and protrusion, pseudopod generation, or actin polymerization. The receptor occupancy only modulates the positive feedback that maintains the leading pseudopod. Secondly, there is no signal processing—each point on the cell's surface is modulated by the local attractant concentration, without reference to points elsewhere in the cell.

Thirdly, receptor adaptation is not required for effective chemotaxis up a static attractant gradient, even at fairly high receptor occupancy, as long as there is a significant difference in the proportion of occupied receptors across the cell. Parts of the edge that lack pseudopods do not gain them when the overall occupancy increases, because positive feedback of the activator is negligible when activator levels are near zero. For adaptation to be dispensable contradicts most current opinion but is supported by several articles, including those showing non-adaptation of movement to high stimuli [38] and lack of adaptation at the G-protein level as measured by FRET [39]. Adaptation at some levels occurs biologically and is required for conditions such as chemotaxis towards sources of biological waves. It was nonetheless surprising that the model would support simple chemotaxis up a linear gradient without adaptation.

The basic motile behaviour of the cell is not fundamentally changed by the chemoattractant. As observed in real cells chemotaxing in moderate gradients [6] but in disagreement with many compass-based explanations for chemotaxis, the rate of pseudopod generation and orientation of new pseudopods are only slightly changed by the chemoattractant.

As the steepness of the attractant gradient applied to the model increases, the accuracy of chemotaxis increases, exactly as seen in real cells (Figure 4C). More surprisingly, however, with steep gradients the model undergoes a qualitative change that precisely resembles real cells. While in low gradients cells nearly always turn from the front, by biasing the behaviour of leading pseudopods, in high gradients they frequently generate a new pseudopod directly towards the attractant source [40]. The model replicates this behaviour (Movie S2; Figure 4D), which was unexpected because we had believed it to be driven by an alternative mechanism. This suggests that pseudopod-centred models can account for the mainstream data supporting signal-induced pseudopods, given steep enough gradients.

When chemotactic cells are presented with a sudden, global change in attractant levels, they respond in a well-defined way. First actin polymerizes all around the cell perimeter, then the cell rounds up as the new F-actin is depolymerized—the “cringe” response [41]—which is followed by repolarization, formation of new pseudopods, and a second peak of actin polymerization (Movie S3). Because this behaviour is consistent and tractable it has been widely used as an assay for chemotactic signal transduction, and the second peak in F-actin in particular has been attributed to a downstream response to PI 3-kinase activation [41]. When the computational model was subjected to a similar sudden increase in receptor occupancy, with the addition of an exponential decay function representing adaptation, the perimeter behaved in a similar fashion to the experimental observations (Movie S4; Figure 4E). Activator levels—corresponding to polymerization of actin filaments—rose rapidly, and modelled cells rounded up, followed shortly afterwards by a drop in activator levels as the inhibitors responded. The time for cells to recover is defined by the rate of adaptation, not by the feedback loops. Strikingly, however, a second complex activator peak occurred that strongly resembles the second experimentally observed F-actin peak (Figure 4F).

This provides an alternative mechanistic explanation for the generation of multiple F-actin peaks. Instead of two pathways with different signal propagation times, as previously predicted [41], the multiple peaks in the model are caused by damped oscillation of a single pathway following a sudden displacement. In this explanation, mutants that mostly lack a second F-actin peak [42] do so because of inefficient positive feedback at the pseudopod level, not separate signalling pathways with different dynamics.

Two further observations support the appropriateness of the pseudopod-centred computational model. Firstly, movement and chemotaxis are relatively robust. The parameters we use (Table S1) are mainly taken directly from Meinhardt [20] and did not need optimization to produce biologically plausible behaviour. Two-fold changes in most of the parameters make only minor, quantitative differences to the behaviour of the simulated cells (Figure S1); indeed many of the single changes shown appear to make chemotaxis more efficient than in our standard conditions. Interestingly, the parameters that were most sensitive to alteration concerned the production of the local inhibitor; changes in the production or decay rates *b_c_* and *r_c_* resulted in either slower migration or repeated movements that are inconsistent with random migration. Raising the diffusion coefficient of the activator (*D_a_*) caused similar problems with repetition, but these could be compensated by corresponding rises in the diffusion coefficient of the local inhibitor (*D_c_*). Secondly, the model handles noise very effectively. Even when the contribution of noise is far greater than the signal from a shallow gradient, chemotaxis is efficient; in shallow gradients, chemotaxis is most efficient over a substantial background of noise (Figure S2). Robustness and tolerance to noise are central to chemotaxis in real cells [43].

### Mechanisms Underlying Eukaryotic Chemotaxis

In compass models of chemotaxis, cells first identify the direction of the attractant gradient, then generate new pseudopods if the cell's direction needs correcting [2]. However recent work suggests that pseudopods do not steer cells this way in shallow gradients. Instead at least two mechanisms act concurrently, both based on the tendency of new pseudopods to be made by bifurcation of existing ones. In the first, new pseudopods are made without requiring external guidance, but cells preferentially retain ones that point in the correct direction [6]. In the second, new pseudopods are generated in stereotypical directions by bifurcation, but their orientation is biased by the direction of the gradient, leading to accurate steering after a number of slight turns [7]. Both mechanisms act concurrently in real cells, though either would be sufficient for chemotaxis alone. We therefore examined the steering of simulated cells to determine whether each of these mechanisms was used.

**Figure 5:**
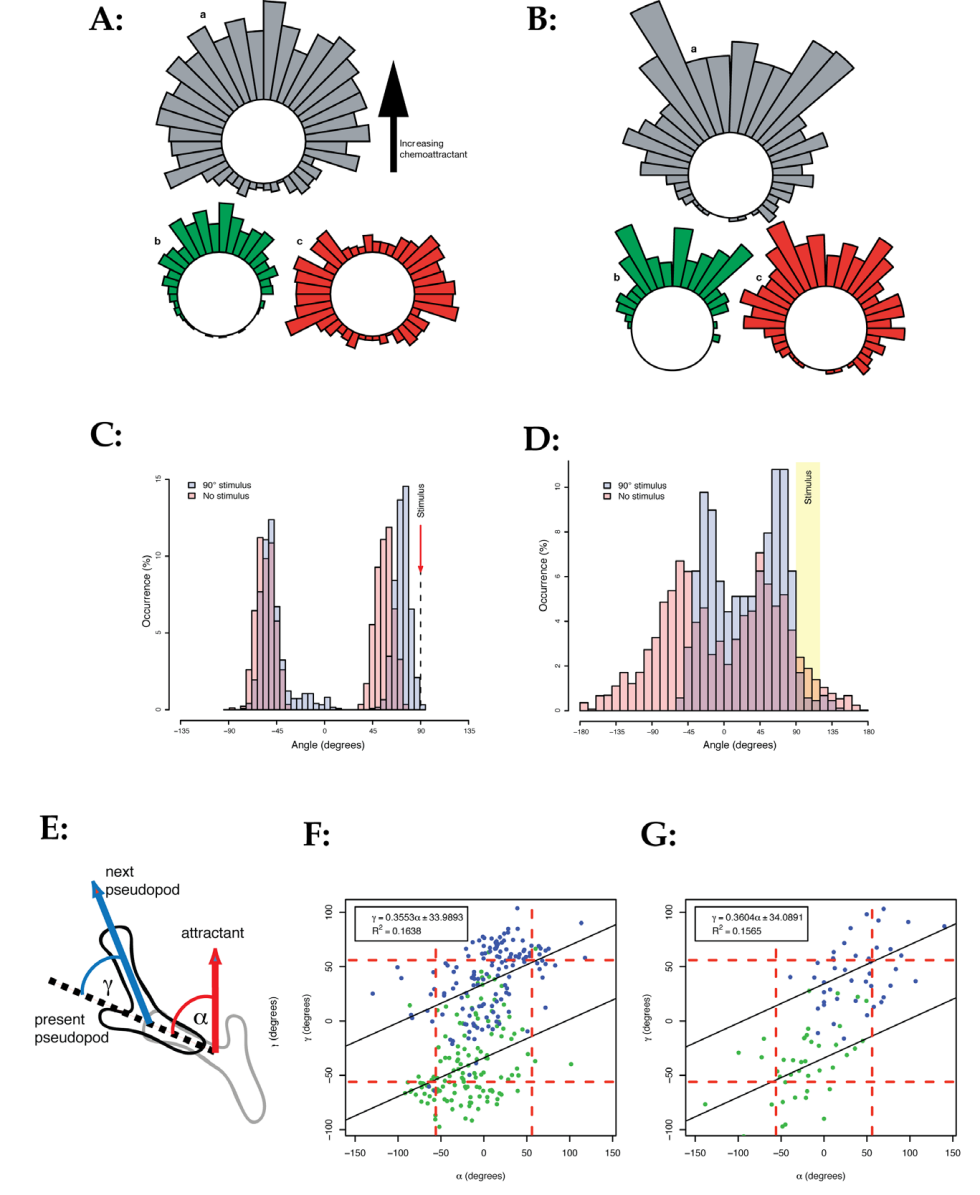
Mechanisms that drive eukaryotic chemotaxis. (A, B) Pseudopod selection from simulated cells (A) and real cells (B; data from [6]). Grey bars represent the total number of pseudopods made in each direction; green bars represent pseudopods that go on to split; red bars represent pseudopods that are retracted without further splitting. (C, D) Signal-driven bias of pseudopod orientation in simulated (D) and real (E) cells (from [7]). Stimulus at +90° causes pseudopods on the same and opposite sides of cell to reorient slightly towards the attractant source. (E, F, G) Bias of pseudopod angle by stimuli from different orientations. (E) shows a schematic showing the angle of the attractant relative to the current pseudopod (α) and the angle of the next pseudopod relative to the current pseudopod (γ). (F) Relationship between α and γ in simulated cells; (G) relationship between α and γ in real cells. Pseudopods made clockwise relative to the previous one are shown in blue, and those made anti-clockwise are shown in green. Dashed red lines represent the positions of pseudopods in the absence of a signal (horizontal) and the angle of attractant that does not alter the pseudopod split-angle (vertical). Black line and equations show line of best fit.

As discussed previously, our computational model generates new protrusions, by bifurcating existing ones, and retracts others [6]. For the analysis of pseudopod selection, we simulated migration in a moderate gradient (initially 5.3–6.6 nM across a cell; shallower gradients give similar but less emphatic results). The point at which each pseudopod splits was identified using a peak detection algorithm, and the daughters followed until one was retracted. We then measured the initial angle of each new pseudopod relative to the direction of the attractant gradient. Figure 5A shows that the simulated cells use selection like real cells (see Figure 5B, data from [6] Figure 3A)—new pseudopods that are pointing well away from the correct direction are nearly always retracted, while pseudopods that point up-gradient are more likely to be retained. The pseudopod selection mechanism is therefore operating for simulated cells as in live cells.

The only obvious difference between the simulated and real data is in the distribution of new pseudopod directions—the “rabbit ears” in the real cells are caused by unequal bifurcation in which the smaller pseudopod points off to one side. Simulated cells bifurcate symmetrically, and thus the distribution of new pseudopods is more even.

The second measured pseudopod-centred mechanism of chemotaxis is directional bias. During bifurcation, new pseudopods can only be generated in a narrow range of angles either side of the parent. However, the mean orientation of new pseudopods is biased slightly towards the attractant source [7]. This makes the path qualitatively similar in the presence or absence of an attractant, but biases accumulate over time and steer the cell. To test whether our simulated cells used this mechanism, we repeatedly reoriented the stimulus as the cell turned (Movie S5). This caused the cell to move in circles. Figure 5C shows that pseudopod bias in the modelled cells is similar to the experimentally measured bias (Figure 5D, replotted from [7]). The mean position of new pseudopods was biased about 15° towards the stimulus. Note that (as in real cells) pseudopods are biased whether they steer the cell towards or away from the stimulus, emphasising that the timing and general location of pseudopod production are not altered by the stimulus. Rather, in both simulation and reality, cell-autonomous processes control the rate and general site at which pseudopods are made and the general area they emerge, and chemoattractant signalling fine-tunes this behaviour.

For a more detailed analysis of how bifurcations are affected by the attractant gradient, we simulated inaccurate migration in shallow gradients and counted a large number of bifurcations. We then measured the angle between the dominant pseudopods before and after the split (schematic, Figure 5E). In simulations run with zero external stimulus, the mean change in the absence of signal is about 55°. We then measured how this angle varied for cells migrating at different angles relative to the attractant gradient. When simulated cells were moving towards the attractant source, the mean change dropped (Figure 5F) to about 30°; as the angle between cell and attractant gradient increased, the mean angle between successive dominant pseudopods also increased by a ratio of about 1° of pseudopod per 3° of additional orientation away from the chemoattractant. At 70° between the gradient and the new pseudopod, the mean angle between successive dominant pseudopods was not altered. Thus the model predicts that the pseudopod split angle is smoothly biased by the attractant direction, in a way that partially compensates for the tendency of new pseudopods to direct the cell away from the attractant and which will steer the cell towards the attractant source over a number of turns.

To compare the simulations with real cells, we examined the data generated by quantitation of movies of cells turning in shallow gradients (Figure 5G; new data, extracted from the same data set as examined in [7]). Again, the correlation between simulated and real data is surprisingly good. Our model thus predicts new data as effectively as it recreates the multiple known features of migration and chemotaxis described previously.

## Discussion

### Biological Correlates

As discussed earlier, we used an undefined model because the large number of incompletely defined pathways makes them require multiple biologically improbable presumptions. Furthermore, many pathways that were thought essential turn out to be dispensable for chemotaxis [44],[45]. However, all of the components required to drive the simulation have physiological equivalents. As discussed earlier, the core activating term corresponds to actin activation, most likely through the Arp2/3 complex. At least three positive feedback loops of the type we use have been described—direct autocatalysis of actin, actin polymerization generating templates for Arp2/3 complex activation, and actin activation of PI 3-kinase.

### Conclusions

Our pseudopod-centred mechanism efficiently couples gradient sensation to migration, overcoming a long-term problem with chemotaxis models [46]. The similarity between the behaviour of modelled and real cells is astounding, especially given the conceptual simplicity of the model and the robustness of the model to changes in parameters. Two apparently separate mechanisms of chemotaxis—pseudopod selection and orientation bias—both emerge from the same simple model, and the complex patterns of actin polymerization and depolymerization following sudden stimuli are also clearly observed without multiple signalling pathways. This emphasises that much of the described complex behaviour of cells is likely to be an emergent property derived from relatively simple pathways.

This implies that future understanding of chemotaxis will require a change in experimental approach. Current research often focuses on how external signals are amplified and processed, and separately on pathways that initiate new actin and new pseudopods. The success of our pseudopod-centred model suggests that a greater emphasis on the physiological mechanisms of pseudopod evolution, and how chemoattractants modulate them, will yield greater fundamental insight.

## Methods

### Numerical Methods

A complete description of the numerical methods used is far beyond the scope of this article and is fully presented in reference [25]. In brief, equations (1)–(3) in Figure 2B are approximated on the evolving cell perimeter using an Arbitrary Lagrangian Eulerian surface finite element method using piecewise linear elements. Time integration is achieved using a semi-implicit approach. The computed activator profile is used to drive a mechanical model of the protrusive and retractive forces exerted on the cell membrane. Movement of the cell is obtained using a level set method and a moving Cartesian mesh. Calculations are performed using the level set toolbox in MATLAB [24].

The fourth equation in Figure 2B, defining the signal, is different from Meinhardt's [20]. In the original Meinhardt model, the location on the cell membrane with the highest receptor occupancy is used—without specification of how it is computed—to centre an assumed sinusoidal variation of the external signal. That model, unlike ours, therefore bypasses a key question in chemotaxis. Instead we relate the signal to the local proportional receptor occupancy, with additional random terms representing noise in the pseudopod system and in the receptor signalling system.

### Cell Methods

The cells in Figure 3D are Dictyostelium AX3 cells, developed for 4 h and imaged exactly as described in [6]. For fluorescence microscopy, similar cells were transfected with an extrachromosomal vector expressing GFP-lifeact and imaged using an Olympus confocal microscope with a 60×1.4 NA objective.

Pseudopod angles were measured using Quimp3 [8] from the same dataset that was used in [7].

## Supporting Information

Figure S1 Robustness of the model. Chemotaxis up a moderate gradient (approximately 5.3 nM to 6.5 nM across the cell) was simulated 10 times. For each parameter in turn, simulations were run at the base value and with the parameter either halved or doubled. In most cases, the chemotactic ability of cells was not qualitatively affected. In a few cases (e.g., doubling of *b_c_* or *D_a_*) the simulations decayed into repetitious changes that did not allow cell movement.
(0.62 MB PDF)

Figure S2 Effects of noise on chemotaxis. Tracks of several different simulated cells, corresponding to the gradients shown. The accuracy increases as the gradient steepens but is also optimal at intermediate or even high noise levels.
(0.71 MB PDF)

Movie S1 Random migration of simulated cells in the absence of attractant followed by moderate attractant gradient. Evolution and migration of the simulated cell from an initially symmetrical state is followed at the indicated time by a gradient from 0 (bottom) to 16 nM (top) across the field.
(1.70 MB MOV)

Movie S2 Chemotaxis to steep attractant gradients. Gradient represents occupancy change from 0% to 20% across the cell.
(0.94 MB MOV)

Movie S3 “Cringe” response to sudden, homogenous rise in attractant concentration. Dictyostelium cells transfected with GFP-lifeact were allowed to migrate randomly without stimulus, then cAMP was suddenly and globally added, causing a sudden redistribution of actin to the cell perimeter.
(2.18 MB MOV)

Movie S4 “Cringe” response in real cells. Modelling the response shown experimentally in Movie S3. Receptors were suddenly and globally upshifted from zero to complete occupancy, using an exponential decay function to simulate adaptation. Background shows arrival and decay of perceived stimulus.
(1.73 MB MOV)

Movie S5 Chemotaxis to a constantly repositioned gradient. Simulated cells were allowed to chemotax to a gradient initially set from 5.3 nM to 7.0 nM across the cell. To maintain a lateral bias, every 200 frames the stimulus was reoriented to +90° from the current direction as indicated by the green wedge.
(2.35 MB MOV)

Table S1 Parameters used. The parameters were mostly taken directly from [20], with a small number of changes needed to counteract the diluting effect of the perimeter expanding at the leading edge.
(PDF)
