# Supplementary figures and images for: Chemotaxis: A Feedback-Based Computational Model Robustly Predicts Multiple Aspects of Real Cell Behaviour
Source: PLoS Biol. 2011 May 17;9(5):e1000618. doi: 10.1371/journal.pbio.1000618 (PMC3096608; doi:10.1371/journal.pbio.1000618)

Neilson et al. Supplementary Figure 1.

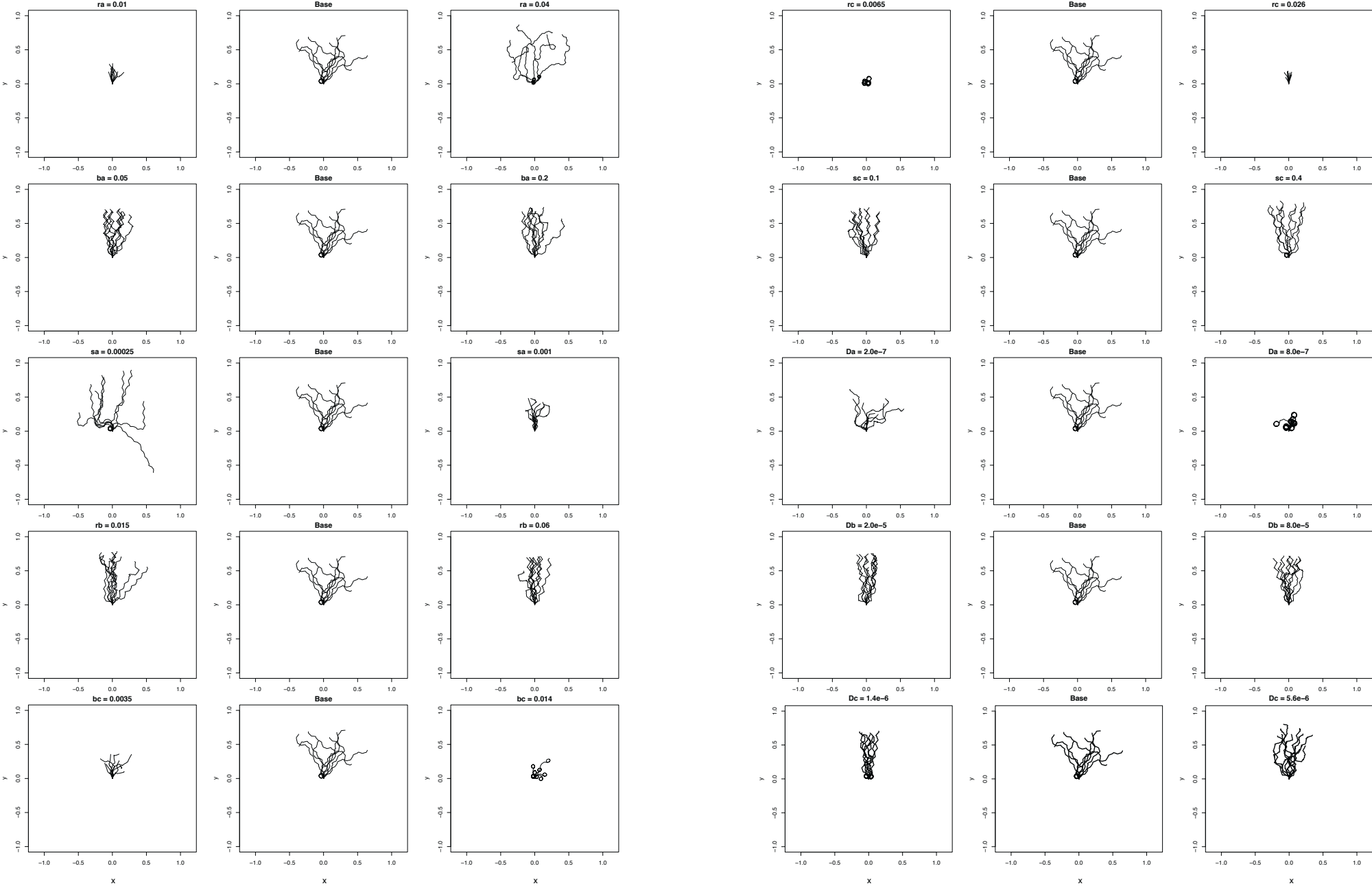

Supplement: Figure S1 — Robustness of the model. Chemotaxis up a moderate gradient (approximately 5.3 nM to 6.5 nM across the cell) was simulated 10 times. For each parameter in turn, simulations were run at the base value and with the parameter either halved or doubled. In most cases, the chemotactic ability of cells was not qualitatively affected. In a few cases (e.g., doubling of bc or Da) the simulations decayed into repetitious changes that did not allow cell movement. (0.62 MB PDF) [file pbio.1000618.s001.pdf]

# Neilson et al. Supplementary Figure 2.

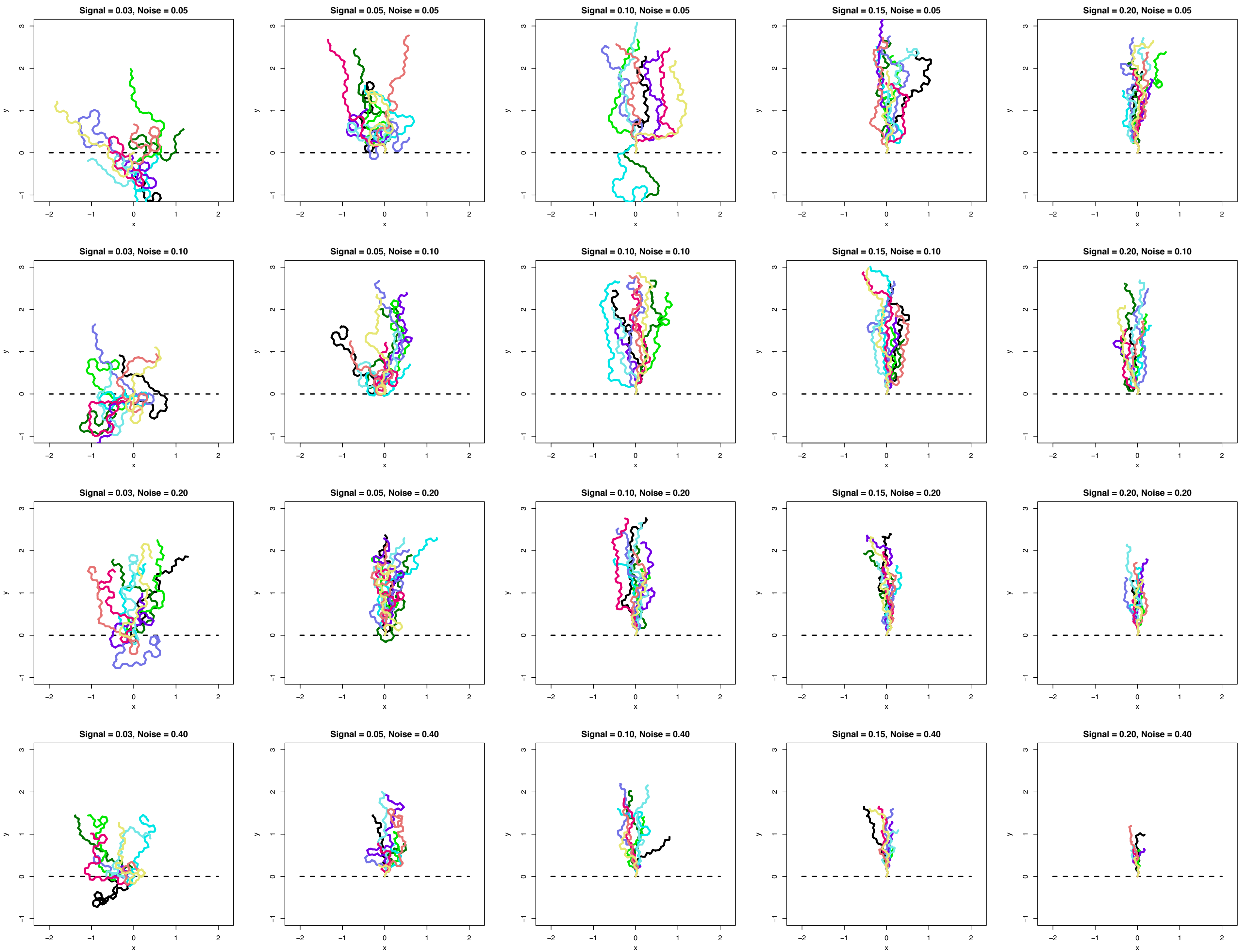

Supplement: Figure S2 — Effects of noise on chemotaxis. Tracks of several different simulated cells, corresponding to the gradients shown. The accuracy increases as the gradient steepens but is also optimal at intermediate or even high noise levels. (0.71 MB PDF) [file pbio.1000618.s002.pdf]
