# Supplementary material for: Chemotaxis: A Feedback-Based Computational Model Robustly Predicts Multiple Aspects of Real Cell Behaviour
Source: PLoS Biol. 2011 May 17;9(5):e1000618. doi: 10.1371/journal.pbio.1000618 (PMC3096608; doi:10.1371/journal.pbio.1000618)
Supplement: Table S1 — Parameters used. The parameters were mostly taken directly from [20], with a small number of changes needed to counteract the diluting effect of the perimeter expanding at the leading edge. (PDF) [file pbio.1000618.s008.pdf]

Table 1: Default values for the parameters that are used in simulations.

Quantity	Symbol	Value
Decay rate of activator†	r_a	2×10^{-2}
Basic production rate of activator†	b_a	1×10^{-1}
Saturation of activator autocatalysis	s_a	5×10^{-4}
Diffusion coefficient of activator	D_a	4×10^{-7}
Production & decay rate of global inhibitor†	r_b	3×10^{-2}
Diffusion coefficient of global inhibitor	D_b	4.0
Production rate of local inhibitor	b_c	7×10^{-3}
Decay rate of local inhibitor†	r_c	1.3×10^{-2}
Diffusion coefficient of local inhibitor	D_c	2.8×10^{-6}
Michaelis-Menten constant†	s_c	2×10^{-1}
Random fluctuation	dr	0.2
Scaling of protrusive velocity	K_{prot}	1×10^{-5}
Starting cortical tension	λ_0	2×10^{-6}
Cortical tension decay constant	β	1×10^{-6}
